# Biofilm-mediated antimicrobial resistance among meat-borne pathogens in Al-Suwaria, Iraq: A cross-species investigation from retail markets

**DOI:** 10.14202/vetworld.2025.2487-2498

**Published:** 2025-08-30

**Authors:** Manal H. G. Kanaan, Zena Kassem Khalil, Ahmad M. Tarek

**Affiliations:** 1 Department of Nursing, Technical Institute of Suwaria, Middle Technical University, Baghdad, Iraq; 2 Department of Optometry, Medical Technical Institute Al-Mansor, Middle Technical University, Baghdad, Iraq; 3 Department of Crime Evidence, Institute of Medical Technology Al-Mansour, Middle Technical University, Baghdad, Iraq

**Keywords:** antimicrobial resistance, *Arcobacter*, Biofilm, *Campylobacter*, Iraq, methicillin-resistant *Staphylococcus aureus*, retail meat, *Salmonella*

## Abstract

**Background and Aim:**

Biofilms formed by foodborne pathogens represent a significant threat to public health by enhancing microbial survival and facilitating antimicrobial resistance (AMR). In Iraq, data on the biofilm-producing potential of key meat-borne pathogens remain scarce, particularly for fastidious organisms such as *Campylobacter, Arcobacter*, and *Salmonella* serovars. This study investigated the prevalence and intensity of biofilm formation in selected meat-borne bacterial isolates and examined their correlation with phenotypic AMR, focusing on moderate to strong biofilm producers.

**Materials and Methods:**

A total of 44 bacterial isolates – including *Staphylococcus aureus* (methicillin-resistant *S. aureus* [MRSA]), *Arcobacter butzleri, Arcobacter cryaerophilus, Campylobacter jejuni, Campylobacter coli, Salmonella enterica* serovars Enteritidis, and *Salmonella* Typhimurium – were recovered from retail meat samples collected between 2018 and 2023 in Wasit, Iraq. Biofilm-forming ability was quantified using microtiter plate assays and interpreted per Stepanovic’s criteria. Antimicrobial susceptibility was assessed through the Kirby–Bauer disk diffusion method, with resistance patterns statistically analyzed for associations with biofilm strength.

**Results:**

Among all isolates, 25% were strong and 40.91% moderate biofilm producers. *Salmonella* serotypes showed the highest biofilm strength (100%), followed by *C. jejuni* (75%) and MRSA (57.14%). A significant correlation (p ≤ 0.05) was observed between biofilm production and resistance to vancomycin, ofloxacin, gentamicin, enrofloxacin, and cefoxitin. Gram-negative isolates with strong to moderate biofilm capacity exhibited resistance rates ranging from 61.90% to 95.24%, while Gram-positive MRSA showed higher resistance to fluoroquinolones and aminoglycosides.

**Conclusion:**

Biofilm production significantly contributes to increase AMR among meat-borne pathogens, compromising food safety and treatment efficacy. Enhanced surveillance, targeted biofilm control strategies, and molecular studies are crucial to mitigate the rising threat of biofilm-associated AMR in the food chain.

## INTRODUCTION

Foodborne infections pose a persistent risk to human health [1]. Biofilms formed by food-borne microorganisms complicate food safety and hygiene practices [2]. Among microbial survival strategies, biofilm production is a key adaptive mechanism in response to environmental stress [3]. Scientists increasingly agree that biofilms represent the predominant mode through which microbes survive and propagate under harsh conditions [4]. These structures protect bacteria from ultraviolet radiation, extreme pH levels, high temperatures, salinity, pressure, nutrient scarcity, and antibiotic exposure, acting as “protective clothing” [5]. Biofilms serve as self-defensive communities that facilitate bacterial proliferation by providing favorable microenvironments and enabling intercellular communication and nutrient sharing [6].

From a contamination standpoint, biofilms are among the leading causes of persistent food safety issues. Their strong adherence to surfaces and food-contact equipment poses a long-term challenge [7]. Biofilms formed by pathogenic and spoilage bacteria act as microbial reservoirs, with a high likelihood of contaminating food and raw materials during processing. This can lead to significant food waste and economic losses for producers [8]. Moreover, the biofilm-forming ability of foodborne pathogens amplifies the risks of foodborne illnesses, posing severe public health threats and economic repercussions [4]. Biofilms remain a critical concern across various sectors of the food industry, including dairy, seafood, meat, and fresh produce processing [4].

Key pathogens of concern in meat processing include *Campylobacter* spp. [7], *Staphylococcus aureus* [9], *Arcobacter* spp. [10], *Escherichia coli* O157:H7 [11], *Salmonella enterica* [12], *Acinetobacter baumannii* [13], and *Listeria monocytogenes* [14]. The ability of these bacteria to persist in environments associated with food production and sanitation increases the likelihood of food poisoning and product recalls [15].

Antimicrobial resistance (AMR) refers to the ability of bacteria and other pathogens to develop mechanisms that render antimicrobial treatments less effective [16]. AMR is a global challenge responsible for millions of deaths annually and poses a serious threat to public health, innovation, and food security [17]. The misuse and overuse of antibiotics in both healthcare and agricultural settings have accelerated the decline in antibiotic efficacy and contributed to increased mortality rates ([18]. Due to the alarming global impact of AMR, it has been termed the “Silent Pandemic,” with projections estimating up to 10 million deaths/year by 2050 if no action is taken [19].

Biofilm-forming bacteria exhibit 100–1000 times greater resistance to antimicrobials compared to their planktonic counterparts [20]. This elevated resistance may be attributed to the protective extracellular matrix of the biofilm and other structural or metabolic factors. The enhanced resistance of biofilms to conventional treatments underscores the urgent need for novel and effective biofilm control strategies [20].

Despite mounting evidence of AMR and biofilm formation among foodborne pathogens globally, there remains a significant paucity of research on this association in Iraq, particularly within the context of meat-borne pathogens. While studies have documented the prevalence of *Campylobacter, Salmonella, Arcobacter*, and methicillin-resistant *S. aureus* (MRSA) in retail meats [7, 9, 10, 12, 21, 22], few have examined the relationship between their biofilm-forming capabilities and antibiotic resistance profiles. Additionally, fastidious organisms such as *Arcobacter* and *Campylobacter*, known for their complex growth requirements and underestimated role in biofilm-associated infections, have received limited attention. No comprehensive investigation has yet explored how biofilm intensity correlates with multidrug resistance across multiple species isolated from meat sources in the Iraqi retail context.

This study aimed to investigate the capacity of selected meat-borne bacterial pathogens – *S. aureus* (MRSA), *Campylobacter* spp.*, Arcobacter* spp., and *S. enterica* serovars – to form biofilms and assess how their biofilm production levels influence AMR. By quantifying biofilm formation and analyzing resistance profiles across strong, moderate, and weak biofilm producers, this research seeks to elucidate the extent to which biofilm development contributes to AMR, thereby informing food safety risk management strategies and antimicrobial stewardship in the Iraqi meat supply chain.

## MATERIALS AND METHODS

### Ethical approval

Medical and Health Research Ethics/Middle Technical University approved this research (MEC NO. 87). No human or animal subjects were used in this investigation. All processes were completed according to approved norms.

### Study period and location

This study was conducted from October 2024 to January 2025 in Al-Suwaria, a city within the Wasit government in the Middle East of Iraq.

### Bacterial isolates

An overall number of 44 bacterial isolates belonging to six different bacterial spp. (*S. aureus, A. butzleri, Arcobacter cryaerophilus, Campylobacter jejuni, Campylobacter coli, S*. Enteritidis, and *Salmonella* Typhimurium) were used in this study. These isolates were isolated between 2018 and 2023 from different meat samples retailed in Wasit markets between 2018 and 2023. Moreover, these bacteria were chosen because of the scarcity of studies on them in Iraq, including *Campylobacter, Salmonella*, and *Arcobacter*, with only two papers available on *Arcobacter*. Consequently, it is crucial to examine these bacteria and their capacity to form biofilms because of their association with antibiotic resistance. Biofilm production was examined in all frozen isolates; however, 44 isolates were selected depending on the type of biofilm generated and the origin of isolates, as various isolates were chosen from each investigation.

### MRSA isolation and identification

MRSA isolates were isolated and identified as described previously by Kanaan [9] and Kanaan and Al-Isawi [22], in which 10 g chunks were put in a sterile plastic bag with 90 mL tryptone soya broth (TSB, Oxoid, CM0129) along with 0.6% yeast extract (YE, Oxoid, LP0021B) (TSB-YE) and then homogenized in a stomacher for 3 min. Five milliliters of homogenate were extracted from 50 mL of TSB. To incubate the culture overnight at 37°C, 20 μL had been plated on Baird-Parker agar (Oxoid, CM1127, Hampshire, UK) with Egg Yolk Tellurite after 18 h at 35°C. Gram staining, mannitol fermentation on mannitol salt agar (Oxoid, CM0085), horse blood agar hemolysis, catalase activity, and rabbit and human plasma coagulase assays were used to identify the organism. The RapID™ Staph Plus System (Remel, R8311009, Kansas, USA) validated *S. aureus* species identification. Dry Spot Staphytect Plus (Oxoid, DR0100M), a latex agglutination test for aggregation factor, protein A, and specific polysaccharides, identified MRSA isolates. The isolates were identified again using the PBP2’ Test Kit (Oxoid, DR0900A), a latex agglutination test for early *S. aureus* PBP2a detection.

### *Arcobacter* spp. isolation and identification

To separate and identify *Arcobacter* spp., 25 g of each sample was mixed for 2 min in a stomacher with 225 mL of sterilized BPW (Oxoid CM0509). We added 25-mL double-strength *Arcobacter* broth (Oxoid, CM965) to 25 mL of the homogenate. For 2 days, an Oxoid AG25 anaerobic jar with CampyGen atmosphere packs (CN0025A, Hampshire, UK) was used to micro-aerobically incubate this suspension at 30°C. After enrichment, 200 μL of each sample was placed in two selective agars: tryptone soy agar (Oxoid, CM0131) with 5% lacked horse blood (SR0048C) and cefoperazone amphotericin teicoplanin improvement and modified charcoal cefoperazone deoxycholate agar (Oxoid, CM739) through Sartorius 0.45-μm-hole nitrocellulose film strainers. After incubating the plates aerobically at 30°C for ~1 h, the filter was removed. The filtrate was uniformly dispersed on two selective agar plates and cultivated under oxygen at 30°C for 2 days. Further biochemical studies were conducted using the Oxoid Biomedical Identification System (OBIS) Campy (Oxoid, ID0803M) to distinguish *Campylobacteraceae* from other Gram-negative organisms. The bacterial species were validated by PCR.

### *Campylobacter* spp. isolation and identification

*Campylobacter* spp. were isolated and identified by weighing 20 g of meat into a sterile stomacher bag, adding 180 mL of Preston enrichment broth, and stomaching for 2 min. After 18 h of selective enhancement at 42°C, 20 μL of enrichment broth was streaked onto mCCDA plates with mCCDA antibiotic (Oxoid, SR155) and raised in an anaerobic environment at 42°C for 72 h under microaerophilic conditions (O_2_ 5%, CO_2_ 10%, N_2_ 85%). Further identification was performed using biochemical reactions (wet mount slide test, oxidase activity, and microaerobic growth at different temperatures). The BioMérieux API^®^ identification kit API CAMPY (Biomerieux, 20800, Marcy-l’Étoile, France) was used to identify thermotolerant *Campylobacter* [23].

### *Salmonella* spp. isolation and identification

As previously stated by Kanaan [12], *Salmonella* spp. was isolated and identified. Briefly, 450 mL TSB with 0.6% YE (TSB-YE) and 50 g of each sample were crushed in a stomacher for 2 min. The mixture was incubated for 18 h at 37°C. Finally, 9 mL of tetrathionate broth (Oxoid, CM0029) and 1 mL of pre-enrichment culture were thoroughly mixed and incubated at 37°C for 18 h. On Xylose Lysine Deoxycholate (Oxoid, CM0469) and Brilliant Green agar (Oxoid, CM0263), a loopful of culture was cultured at 37°C for 1–2 days. Oxoid biochemical identification system (ID0570) was used to distinguish *Salmonella* spp. from other organisms with similar colonial appearance on a selective *Salmonella* medium. mPCR confirmed *S*. Enteritidis and *S*. Typhimurium.

### Culture conditions

#### MRSA

An overall number of 14 frozen single-use stocks of MRSA obtained from previous studies by Kanaan [9], and Kanaan and Al-Isawi [22] were preserved at −80°C in a double-strength TSB-YE with 20% medical glycerin, thawed at 4°C for 18 h, subcultured onto TSA, and then, incubated overnight aerobically at 35°C–37°C.

#### Arcobacter species

A total of 10 frozen single-use stocks of *Arcobacter* isolates (eight *A. butzleri* and two *A. cryaerophilus*) obtained from a previous study by Kanaan [10] were subjected to a biofilm production assay by defrosting them at 4°C and rehydrating them on mCCDA. Subsequently, they were maintained in an aerobic incubator at 30°C for 2 days.

#### Campylobacter species

A total of 14 frozen single-use stocks of *Campylobacter* isolates (eight *C. jejuni* and six *C. coli*) obtained from a previous study by Kanaan and Abdulwahid [23] were subjected to a biofilm production assay. Originally kept in a double-strength nutrient broth (Oxoid, CM0001) with 20% pure medical glycerin at a temperature of −80°C, the bacterial stocks were allowed to thaw overnight in a refrigerator. Following a microaerophilic environment for 24 h in an anaerobic container using a Campy Gen packet, the stocks were subsequently subcultured on mCCDA-Preston agar (Oxoid, CM0739).

#### S. enterica serovars Enteritidis and S. Typhimurium

Six frozen single-use stocks of *S. enterica* isolates (four *S*. Enteritidis and two *S*. Typhimurium) obtained from a previous study by Kanaan [12] were subjected to a biofilm production assay. Stocks that were preserved at −80°C in a double-strength TSB-YE with 20% pure medical glycerin were allowed to defrost in the fridge overnight and then cultivated at 37°C for 18 h in TSA. The isolates were then transferred to TSB devoid of glucose and raised at 37°C for an additional 24 h. An inoculum of 3 × 10^8^ CFU/mL was prepared by adjusting the turbidity of the bacterial suspension in TSB without glucose according to the McFarland standard No. 1 [12].

#### Biofilm production in S. aureus

As previously stated by Yousefi *et al*. [24], a microtiter plate approach had been used to measure the quantity of biofilm development, with slight modifications. Briefly, six to eight well-isolated identical colonies from fresh TSA are suspended in 10 mL of TSB with 0.25% glucose and incubated without shaking for 24 h at 37°C. After incubation, the culture is vortexed, thereafter diluted 1:100 with fresh TSB with 0.25% glucose. The diluted bacteria are vortexed and then diluted cultures were placed in 200 μL wells of sterile 96-well flat-bottom polystyrene tissue culture treatment plates (Sigma-Aldrich, Costar, USA) and underwent incubation at 37°C for 48 h. The negative control wells comprised only 200 μL of TSB - 0.25% glucose deprived of bacterial suspension. Phosphate buffer saline (0.2 mL, pH 7.2) will be added to the wells 4 times for washing. This effectively eliminated any microorganisms that were floating about. Bacterial biofilms that attached to the wells had been preserved with 2% sodium acetate and stained with 0.1% crystal violet (CV). After rinsing the plates with deionized water to remove any remaining discoloration, they were set aside to dry. The stained adherent biofilm’s optical density (OD) has been identified at 570 nm using a micro enzyme-linked immunosorbent assay (ELISA) auto reader (model 680, Biorad). This experiment was conducted three times, each time in duplicate.

#### Biofilm production in Arcobacter species

The quantitative determination of biofilm generation had been performed with microtiter plate technique, with some minor adjustments, as described by Teh *et al*. [25]. *Arcobacter* broth (CM0965B, Oxoid) was used to cultivate 6-8 well-isolated colonies from fresh agar overnight with agitation. The colonies were subsequently diluted until an OD of 0.25 at 600 nm was achieved. A suspension then added to each well of a 96-well microtiter flat-bottom polystyrene plate (Sigma-Aldrich, Costar, USA) using 200 μL of it. The plate was then incubated at 30°C under aerobic as well as microaerobic conditions by use of a light agitation at 30 rpm for 48 h. There was just 200 μL of *Arcobacter* broth in the negative control wells, devoid of any bacterial suspension. Phosphate buffer saline (0.2 mL, pH 7.2) will be added to the wells four times for washing. This effectively eliminated any microorganism that was floating about. Bacterial biofilms that had attached to the wells had been kept intact with 2% sodium acetate then tinted with 0.1% CV. Plates had been dried after deionized water removed excess discoloration. OD of colored adherent biofilm has been achieved through applying micro ELISA auto reader (model 680, Biorad, UK) at wavelength 570 nm. There were three separate runs of the experiment, each of which was carried out in triplicate.

#### Biofilm production in Campylobacter species

CV staining was used for measuring biofilm formation as described previously by Reeser *et al*. [26] for *C. jejuni*, with some modifications; used one culture broth instead of three, wash plates 4 times with phosphate buffer saline to eliminate floating bacteria, then fix biofilm with 2% sodium acetate before staining. To create shaking cultures, cells were taken off the agar plate and added to Mueller-Hinton broth (MHB, Oxoid, CM405B). The mixture had been incubated for 18 h at 37°C in a microaerophilic environment. Using spectrophotometric analysis, the cell culture was standardized to an OD of 0.25 at 600 nm in this broth after overnight growth. To make biofilm development easier, 1 mL of MHB was cultured overnight and then added to the wells of 24-well polystyrene Costar plates (Corning, USA) until the OD (OD_600_ nm) reached 0.025, which is equivalent to around 2.5 × 10^7^ CFU. Triplicate testing was performed on each isolate. The control wells were filled with sterile MHB broth alone. Microaerophilic conditions were maintained during incubation at 37°C to the plates. Following the cultivation period, medium from every well was meticulously extracted. Four times, 0.2 mL of phosphate buffer saline had been used to wash the wells. As a result, floating microbes were eliminated. Following the fixation of biofilms being formed in wells by sodium acetate at a concentration of 2%, they were dried at 55°C for 30 min. After that, they were dyed with CV for 5 min at 24°C. The unbound residue was eliminated by two washes with deionized water. A mixture of 80% ethanol and 20% acetone was used to decolorize the bind CV. The wells became dry at 55°C for 15 min. We used an ELISA auto reader (model 680, Biorad) to spectrophotometrically detect the absorbance at 570 nm. Afterward, 100 μL of the solution was moved from an individual well to a plate with 96 wells. There were three separate runs of the experiment, each of which was carried out in triplicate.

#### Biofilm production in Salmonella serotypes

A microtiter plate approach, which was established by Borges *et al*. [27] was utilized to quantitatively quantify the amount of biofilm generation, with slight modifications; used a single incubation temperature (37°C) rather than four, which is the ideal temperature for *Salmonella* growth, and use phosphate buffer saline instead of water to wash the plates. Resuspend the biofilm in 200 μL of 95% ethanol. In addition, instead of utilizing the EL×800 absorbance reader, record the OD of each well with an ELISA auto reader. To evaluate biofilm formation, three wells of four sterile, 96-flat-bottomed polystyrene plates housed 200 μL of each suspension. Triplicate inoculations of negative control wells with just TSB without glucose. We performed biological triplicates to analyze biofilm. Plates had been covered and incubated aerobically at 37°C for 24 h. To dispose of the contents after incubation, the container was turned upside down and the liquid broth was shaken off. A plate has been then rinsed with phosphate buffer saline solution. To facilitate the removal of detached cells, the washing procedure was done twice. Attached bacteria were fixed with 200 μL methanol for 15 min. The plates were left to incubate at room temperature for 1 h after adding 200 μL of 1% CV dye to every well. Wells then rinsed 5 times with phosphate buffer saline solution after incubation, then dye had been discarded. Laboratory paper towels were used to blot the microtitre plate dry, and then, it was left to dry at room temperature. Each well was then incubated at 24°C for 5 min after 200 μL of 95% ethanol had been added to them. Using a 550 nm wavelength and an ELISA auto reader (model 680, Bio-Rad, USA), the OD of every well was recorded. Each *Salmonella* serotype’s ODs were calculated by averaging three separate measurements taken in triplicate.

#### Interpretation of biofilm production

The interpretation of biofilm production was conducted according to the criteria of Stepanovic *et al*. [28], in which the optical density cut-off (ODC) is the average of the negative control’s ODs plus three times the standard deviation (SD) of those densities. The absorbance of adherent cells stained with CV was used to examine and classify the biofilm development by different isolates. The isolates were classified into four groups based on their biofilm production rates: (o) isolates that do not produce biofilm (ODs ≤ ODC), (+) isolates that produce weak biofilm (ODC <ODs ≤2×ODC), (++) isolates that produce moderate biofilm (2×ODC <ODs ≤4×ODC), or (+++) isolates that produce robust biofilm (4×ODC <ODs). The final OD value of isolate = average OD of isolate - OD c.

ODc = average OD of negative control + (3× SD of negative control).

#### Antibiotic susceptibility test

All those isolates had been screened for antibiotic susceptibility using the Kirby-Bauer disk diffusion method. Results were interpreted in accordance with the Clinical and Laboratory Standards Institute’s guidelines [29]. MRSA screened against 10 antimicrobial agents as described earlier by Kanaan [9] and Kanaan and Al-Isawi [22]. *Arcobacter* isolates were screened against 11 antimicrobials as described by Kanaan [10]. *Campylobacter* isolates from poultry meat were screened to eight antibiotics as described by Kanaan and Abdulwahid [23]. *Salmonella* serotypes were screened to 12 antimicrobials as described previously by Kanaan [12].

### Statisical analysis

MedCalc Software BVBA version 23.02 (BE, USA) was used to compute differences that were statistically significant. A number of descriptive statistics were utilized, including the proportion, the mean, and the SD. For the chosen biofilm-producing bacteria, through means of a two-sample Chi-square test, at 5% significance level to compare percentages MedCalc Software Ltd. [30].

## RESULTS

### Distribution of biofilm-forming isolates among bacterial species

Table 1 of our data revealed that among 44 isolates, *S. aureus* (MRSA) and *Campylobacter* spp. accounted for the vast majority of the microbes linked to biofilm formation (31.82% and 31.82%, respectively), followed by *Arcobacter* spp. (18.18%) and *Salmonella* serotypes (13.64%). *Salmonella* serotypes showed the highest prevalence of strong to moderate biofilm producers (6/6, 100%), followed by *C. jejuni* (6/8, 75%), *C. coli* (4/6, 66.67%), MRSA (8/14, 57.14%), *A. butzleri* (4/8, 50%), and *A. cryaerophilus* 1/2 (50%).

**Table 1:** Effect of biofilm production of some meat-borne pathogens on antibiotic sensitivity based on sample type.

Organism	Biofilm production	Meat type	Sensitivity to the following antibiotics	Number of Abs to which the isolates are resistant/total Abs (%)
MRSA	Moderate	Raw chicken	VAN, OFL	8/10 (80)
MRSA	Moderate	Raw chicken	T, GM, ENF, and OFL	6/10 (60)
MRSA	Moderate	Raw chicken	VAN, T, and ENF	7/10 (70)
MRSA	Weak	Raw chicken	VAN, GM, ENF, and OFL	5/10 (50)
MRSA	Weak	Raw chicken	VAN, FOX, GM, ENF, and OFL	5/10 (50)
MRSA	Strong	Raw chicken	VAN, OFL	8/10 (80)
MRSA	Weak	Frozen chicken	VAN, FOX, T, GM, ENF, and OFL	4/10 (40)
MRSA	Moderate	Frozen chicken	GM, OFL	8/10 (80)
MRSA	Weak	Cattle	VAN, GM, ENF, and OFL	6/10 (60)
MRSA	Weak	Cattle	ME, VAN, OX, T, GM, OFL	4/10 (40)
MRSA	Moderate	Cattle	VAN, ENF	8/10 (80)
MRSA	Strong	Cattle	VAN	9/10 (90)
MRSA	Moderate	Cattle	VAN, GM	8/10 (80)
MRSA	Weak	Cattle	VAN, FOX, GM, ENF, and OFL	5/10 (50)
*A. butzleri*	Moderate	Chicken	FOX, GM, and NOR	8/11 (72.73)
*A. butzleri*	Moderate	Chicken	CIP, FOX, and NOR	8/11 (72.73)
*A. butzleri*	Weak	Chicken	CIP, FOX, GM, NOR, ND, and AMC	5/11 (45.45)
*A. butzleri*	Negative	Chicken	CIP, FOX, VAN, GM, NOR, AMP, ND, and AMC	3/11 (27.27)
*A. butzleri*	Strong	Cattle	CIP, FOX	9/11 (81.82)
*A. butzleri*	Moderate	Cattle	CIP, FOX, VAN, and GM	7/11 (63.64)
*A. butzleri*	Weak	Cattle	CIP, FOX, GM, NOR, and ND	6/11 (54.55)
*A. butzleri*	Negative	Cattle	CIP, FOX, VAN, GM, NOR, and ND	5/11 (45.45)
*A. cryaerophilus*	Moderate	Chicken	CIP, FOX, GM, and NOR	7/11 (63.64)
*A. cryaerophilus*	Weak	Cattle	CIP, FOX, GM, NOR, AMP, ND, and AMC	4/11 (36.36)
*C. jejuni*	Strong	Chicken	ND	7/8 (87.5)
*C. jejuni*	Moderate	Chicken	ND, GM	6/8 (75)
*C. jejuni*	Weak	Chicken	ENR, ND, and GM	5/8 (62.5)
*C. jejuni*	Negative	Chicken	ENR, ND, GM, and T	4/8 (50)
*C. jejuni*	Strong	Chicken	__	8/8 (100)
*C. jejuni*	Moderate	Chicken	ND, GM	6/8 (75)
*C. jejuni*	Strong	Turkey	--	8/8 (100)
*C. jejuni*	Moderate	Turkey	CIP, GM	6/8 (75)
*C. coli*	Strong	Chicken	--	8/8 (100)
*C. coli*	Strong	Chicken	ND	7/8 (87.5)
*C. coli*	Negative	Chicken	CIP, E, GM, ND, NOR, and T	2/8 (25)
*C. coli*	Strong	Turkey	--	8/8 (100)
*C. coli*	Moderate	Turkey	ND, GM, and OFL	5/8 (62.5)
*C. coli*	Negative	Turkey	ND, CIP, OFL, E, T, GM, and VAN	1/8 (12.5)
*S.* Enteritidis	Strong	Raw chicken	GM	11/12 (8.33)
*S.* Enteritidis	Moderate	Raw chicken	AMI, GM, FOX, AMP, CHL, CIP	6/12 (50)
*S.* Enteritidis	Strong	Frozen chicken	CTC	11/12 (8.33)
*S.* Enteritidis	Moderate	Frozen chicken	GM, AXO, CTC, and CIP	4/12 (33.33)
*S.* Typhimurium	Moderate	Raw chicken	FOX, CIP	2/12 (16.67)
*S.* Typhimurium	Moderate	Frozen chicken	AMI, GM, FOX, AMP, CHL, and CIP	6/12 (50)

MRSA=Methicillin-resistant *Staphylococcus aureus*, Abs=Antibiotics, VAN=Vancomycin, OFL=Ofloxacin, T=Tetracycline, GM=Gentamicin, ENF=Enrofloxacin, FOX=Cefoxitin, ME=Methicillin, OX=Oxacillin, NOR=Norfloxacin, CIP=Ciprofloxacin, AMP=Ampicillin, AMC=Amoxicillin/clavulanic acid, ND=Nalidixic acid, E=Erythromycin, CHL=Chloramphenicol, CTC=Cefotaxime/clavulanic acid, AXO=Ceftriaxone, *A. butzleri=Arcobacter butzleri, C. jejuni=Campylobacter jejuni, C. coli=Campylobacter coli, S.* Enteritidis=*Salmonella* Enteritidis, *S.* Typhimurium=*Salmonella* Typhimurium

### Source of biofilm-producing isolates by meat type

Biofilm-producing bacteria were isolated from chicken meat (25%), cattle meat (22.73%), raw chicken meat (20.45%), frozen chicken meat (11.36%), and turkey meat (9.09%). Strong biofilm production was caused by *Salmonella* Enteritidis (50%) and *C. coli* (50%).

### Overall biofilm formation profile

Additionally, based on the biofilm assay, the findings (Table 2) indicated that the number of strong biofilm producers was 11 (25%), moderate biofilm producers were 18 (40.91%), and weak or non-biofilm producers were 15 (34.09%).

**Table 2:** Prevalence of biofilm production isolates based on biofilm type.

Number of isolates	Biofilm formation	Number of isolates (%)
44	Strong	11 (25)
	Moderate	18 (40.91)
	Weak/negative	15 (34.09)

### Association between biofilm production and AMR in MRSA

To determine the correlation between antibiotic resistance and biofilm production, we examined the phenotypic distribution of resistance according to the biofilm formation categories. The results (Table 3) revealed increased antibiotic resistance in isolates with strong to moderate biofilm production compared with isolates that do not produce or produce weak biofilm. In particular, 100% of isolates with strong to moderate biofilm production exhibited resistance to cefoxitin, methicillin, and oxacillin compared to 50%–83.33% of non-to-weak producers’ isolates.

**Table 3:** Correlation between biofilm production by methicillin-resistant *S. aureus* and antibiotic resistance.

Antimicrobial agents	Prevalence of resistance in strong and moderate MRSA-producing biofilms (n = 8) (%)	Prevalence of resistance in negative and weak biofilm-producing *S. aureus* isolates (n = 6) (%)	p-value
Vancomycin	2 (25)	0 (0)	0.0495
Ofloxacin	4 (50)	0 (0)	0.0027
Tetracycline	6 (75)	3 (50)	0.1797
Gentamicin	5 (62.5)	0 (0)	0.0005
Enrofloxacin	5 (62.5)	1 (16.67)	0.0149
Cefoxitin	8 (100)	3 (50)	0.0027
Methicillin	8 (100)	5 (83.33)	0.1171
Oxacillin	8 (100)	5 (83.33)	0.1171
Ampicillin	8 (100)	6 (100)	----
Erythromycin	8 (100)	6 (100)	----
Fusidic acid	8 (100)	6 (100)	----

MRSA=Methicillin-resistant *Staphylococcus aureus, S. aureus=Staphylococcus aureus*

Moreover, the high prevalence of MDR observed for ofloxacin, tetracycline, gentamicin, and enrofloxacin ranged from 50% to 75%, compared to 0%–50% of non-to weak producers’ isolates. A low level of resistance to vancomycin was observed (25%), compared with 0% of non-weak producers. Statistically, the creation of biofilms had a profound impact (p ≤ 0.05) on the resistance of the isolates to vancomycin (p = 0.0495, χ^2^ = 3.857), ofloxacin (p = 0.0027, χ^2^ = 9.000), gentamicin (p = 0.0005, χ^2^ = 12.273), enrofloxacin (p = 0.0149, χ^2^ = 5.928), and cefoxitin (p = 0.0027, χ^2^ = 9.000).

### Antibiotic resistance in biofilm-producing Gram-negative bacteria

Our results (Table 4) reported the same observation with higher antibiotic resistance in strong to moderate biofilm-producing Gram-negative bacteria, ranging from 61.90% to 100%, compared to non-weak biofilm producers.

**Table 4:** Correlation of biofilm production by some Gram-negative food-borne pathogens with antibiotic resistance.

Antimicrobial agents	Prevalence of resistance in Gram-negative isolates with strong and moderate biofilm production (n = 21) (%)	Prevalence of resistance in Gram-negative and weak biofilm-producing isolates (n = 9) (%)	p-value
Ofloxacin	18 (85.71)	9 (100)	0.0331
Tetracycline	20 (95.24)	8 (88.89)	0.5298
Gentamicin	10 (47.62)	0 (0)	0.0127
Cefoxitin	14 (66.67)	4 (44.44)	0.2628
Amoxicillin/clavulanic acid	18 (85.71)	6 (66.67)	0.2401
Ciprofloxacin	12 (57.41)	2 (9.52)	0.0170
Nalidixic acid	16 (76.91)	0 (0)	0.0001
Ampicillin	19 (90.48)	8 (88.89)	0.8959

An interesting observation obtained in this study was that 100% of non-to-weak biofilm producers were resistant to ofloxacin, compared to strong to moderate biofilm-producing Gram-negative isolates (85.71%) exhibiting resistance to it.

Statistically, the formation of biofilms had a profound impact (p ≤ 0.05) on the resistance of the isolates to ofloxacin (p = 0.0331, χ^2^ = 4.540), gentamicin (p = 0.0127, χ^2^ = 6.214), ciprofloxacin (p = 0.0170, χ^2^ = 5.697), and nalidixic acid (p = 0.0001, χ^2^ = 14.495).

### Comparison of resistance patterns between MRSA and Gram-negative isolates

**Figure 1:**
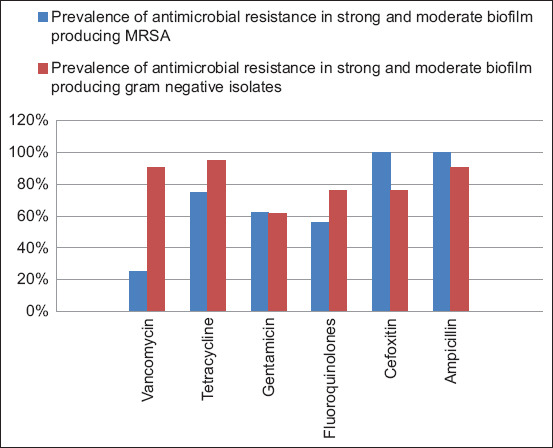
Prevalence of antimicrobial resistance in strong and moderate biofilm-producing meat-borne pathogens.

Moreover, weaker resistance to vancomycin, tetracycline, and fluoroquinolones was found in strong to moderate biofilm-producing MRSA isolates than in strong to moderate biofilm-producing Gram-negative isolates, which showed weaker resistance to beta-lactams (Figure 1).

Additionally, resistance to selected antimicrobials was not statistically influenced by bacterial groups (p ≥ 0.05), except for vancomycin (p = 0.0005).

## DISCUSSION

### Biofilm formation in foodborne pathogens

Microbial biofilms are a serious issue in the food processing sector. Increasing amounts of data point to biofilms helping Gram-positive and Gram-negative bacteria survive in the food chain [31]. Biofilms may consist of one or more microbial species. A single-species biofilm adopts a transient lifestyle, wherein each organism collaborates within a collective to enhance survival under challenging conditions [32]. Multi-species biofilms may comprise more than 500 bacterial taxa, resulting in enhanced resistance and prolonged lifespan compared to single-species biofilms, since microorganisms collaborate and share their unique survival strategies, conferring various protective benefits [5]. In this study, the capacity of some foodborne pathogens isolated from meat samples to form biofilms was investigated. The impact of the biofilm on the ability of the isolates to resist antimicrobials was also evaluated. Our results (Table 1) revealed that among 44 isolates, a significant number of the species that were associated with the formation of biofilm were MRSA (31.82%) and *Campylobacter* spp. (31.82%), whereas *Salmonella* serotypes showed the highest prevalence of strong to moderate biofilm producers (100%). Moreover, our results (Table 2) showed that 25% of the tested bacteria appeared to be strong biofilm producers, 40.91% were moderate, and 34.09% were weak or non-biofilm producers.

### Food industry implications of biofilm-associated contamination

Foodborne pathogenic bacteria attaching to food through biofilms and living on food-contact surfaces can cause contamination during processing, negative effects on product quality and shelf life, and illness spread. These bacteria pose a significant threat to the development of the food industry and human health [4, 33].

### Biofilm-forming potential of *S. aureus*

*S. aureus* ranks high among the most prevalent bacteria that can cause food poisoning in terms of food safety issues [34]. *S. aureus* is a highly adaptable organism that can thrive in different habitats by creating biofilms [35]. This species demonstrates a connection between its biofilm formation and cell adhesion capabilities and the creation of the PIA gene, in addition to the proteins encoded by several genes that assist in the synthesis and elongation of PIA [36].

### Biofilm-mediated resistance in *Salmonella* serotypes

*Salmonella* causes 1.3 billion enteric infections and 500,000 fatal diarrhea-related incidents worldwide [37]. The spread of *Salmonella* strains in Iraq is a serious concern [38]. Since they are linked to many human salmonellosis cases caused by eating animal-contaminated foods, NTS serovars are nevertheless of global health concern [39]. There is minimal data on a link between poultry *Salmonella* serotypes, multiple antibiotic resistance, and biofilm formation [27]. Biofilm-forming *Salmonella* serotypes have been found in food and processing industries in developed and developing countries [40]. *Salmonella* serotypes can produce biofilms that endanger the hygiene and safety of food processing facilities. Food prices may rise due to increased plant sanitation expenses resulting from the presence of this *Salmonella* serotype in food or on surfaces that come into contact with food. In addition, the confirmation of biofilm-producing *Salmonella* serotypes in poultry samples in this study supports the findings of Wang *et al*. [41] in China.

### Contrasting biofilm formation in *Arcobacter* and *Campylobacter*

*Arcobacters* and *Campylobacters* produce biofilms on various surfaces [42]. Nevertheless, *Arcobacter* are often categorized in the literature as ineffective biofilm producers [43], which is consistent with our results. Similarly, other studies by Teh *et al*. [25] and Šilha *et al*. [42] have indicated that *Campylobacters* are not proficient biofilm producers, which contradicts our research results. Previous research by Gunther and Chen [44] indicates that *Campylobacter* species generate a greater quantity of biofilm in their indigenous microaerophilic environment, a phenomenon corroborated in other studies, and heightened biofilm activity has been observed in isolates derived from meat [43]. The first step in biofilm formation is the adhesion of bacteria to the different materials of industrial surfaces [45]. Biofilm development is boosted by associating with the surfaces of foods or their organic components [46]. Remarkably, in retail food samples, *Campylobacter* developed more biofilms with *E. coli* or *Pseudomonas aeruginosa* than with *E. coli* alone [46]. *C. jejuni* may thrive due to numerous chicken-derived bacterial populations in poultry processing factories [47]. The conditioning layer of meat juice on abiotic surfaces provides an adhesive substrate for *C. jejuni* biofilms [47]. Biofouling, the formation of absorbed layers on a surface, may indicate conditioning in adverse settings such as the food chain or pipes [48]. Thus, *C. jejuni’s* best chance of surviving in the food chain is through adhering to surfaces and other species’ biofilms.

### Biofilm formation and its correlation with multidrug resistance

Increased levels of antibiotic resistance were discovered in strong-to-moderate biofilm-producing bacteria compared with non-to-weak biofilm producers (Table 3), in which 100% of strong-to-moderate biofilm-producing MRSA exhibited MDR compared to 50%–83.33% of non-to-weak producers’ isolates. A low level of resistance to vancomycin was observed (25%), compared with 0% of non-weak producers. Previous studies by Eyoh *et al*. [49] and Navon-Venezia *et al*. [50] have found more MDR strains among biofilm producers than non-biofilm formers due to limited treatment options. Bacterial biofilm communities cause persistent infections and antibiotic resistance [51]. These organisms are difficult to eradicate and control because biofilms and bacterial cells in complex matrices might increase antimicrobial and sterilizing agent resistance [52]. The biggest problem with *S. aureus* biofilms is antibiotic resistance and host defense evasion [53]. MDR may be caused by extracellular polymeric molecules in biofilm-forming *S. aureus*. These chemicals block the diffusion of antibiotics, which can alter the molecular transit to the biofilm interior or antimicrobial material reactivity with the matrix material [53].

### Antibiotic resistance in Gram-negative biofilm producers

Our findings (Table 4) revealed that strong to moderate biofilm-forming Gram-negative bacteria had increased antibiotic resistance ranging from 76.2% to 100% compared with non-weak producers, which is consistent with previous results [49, 54]. Biofilm matrices act as physical and chemical barriers that prevent antimicrobials from accessing bacteria, hindering pathogen control, and promoting resistance in biofilm-forming or infected microorganisms [40]. Similar to this work, *Salmonella* infections have an alternate sigma factor RNA polymerase sigma factor (RpoS) along with flagella topologies that enable biofilm development. Thus, *Salmonella* biofilms may hinder antimicrobial therapy for salmonellosis [40]. *Campylobacter* and *Arcobacter* may form biofilms that exhibit increased resistance to antimicrobials and disinfectants, posing a significant challenge in the food industry, particularly in the processing of chicken meat [55].

### Inverse trends in ofloxacin resistance and biofilm formation

Interestingly, 100% of non-to-weak biofilm producers were resistant to ofloxacin, but 85.71% of strong-to-moderate biofilm producers were resistant. Similar findings have been reported in other bacterial species in Egypt [56], Iran [57], and China [58], in which an inverse relationship was found between antibiotic resistance and the capacity to generate biofilm.

### Mechanistic insights: Biofilm-induced resistance and fitness cost

Biofilms impair antimicrobial diffusion by exposing embedded cells to sub-lethal doses of these drugs, which promote biofilm growth. This is called adaptive tolerance, and it develops not from inherited resistance but from metabolic or gene expression changes [59]. The “fitness cost” trade-off is reinforced by the negative correlation between biofilm and resistance determinants, which states that gaining resistance reduces bacterial pathogenicity, transmissibility, or growth rate [56]. As a result, pathogenic bacteria in biofilm communities typically have high virulence and antimicrobial tolerance or resistance, making it possible for them to survive even rigorous antibiotic treatment regimens. This makes microbial biofilm infections tough to treat. This demonstrates the need for new and improved methods of controlling or eliminating pathogenic biofilms [4].

### Strengths and limitations of the study

Meat-borne fastidious bacteria, such as *Salmonella* and *Campylobacter*, are rarely studied in Iraq. We have only two studies on *Arcobacter*, but with no antibiotic restrictions in the country and rising resistance, it is vital to research the origins of this resistance, which is one of the strengths of this study. Due to cost constraints, only one approach was used to create biofilm from the selected pathogens, which require special treatment and specific culture medium. However, our study had some limitations. All screened isolates were recovered from meat samples only. Resistance was not studied genetically but rather based on phenotype. Hence, it is recommended that future research employ a variety of dietary and clinical samples and that molecular identification of genes important for resistance and biofilm formation.

## CONCLUSION

This study demonstrated that biofilm formation is widespread among meat-borne bacterial pathogens in Iraq. Among the 44 isolates tested, *Salmonella* serotypes, *Campylobacter* spp., and MRSA showed a notably high prevalence of strong to moderate biofilm-forming ability. A total of 25% of isolates were strong producers, while 40.91% were moderate producers.

A significant association was observed between biofilm production and AMR. Isolates with strong to moderate biofilm-forming capacity exhibited elevated resistance levels, particularly to cefoxitin, methicillin, oxacillin, gentamicin, and enrofloxacin. Statistical analysis confirmed that biofilm formation significantly contributed to increased resistance profiles, especially in Gram-negative bacteria.

These findings raise serious concerns regarding food safety and public health. The persistence of biofilm-forming, multidrug-resistant organisms on meat and food-contact surfaces may facilitate transmission of resistant infections to consumers, complicate treatment outcomes, and challenge current sanitation protocols in the food industry.

Further studies should include a broader range of clinical and food-related samples to enhance generalizability. Molecular characterization of resistance genes and biofilm-regulating pathways is recommended to deepen understanding of the mechanisms involved. Additionally, research into anti-biofilm strategies and interventions suitable for meat processing environments should be prioritized.

This study emphasizes the role of biofilm formation in enhancing AMR among foodborne pathogens. It highlights the necessity of incorporating biofilm control measures in food safety frameworks and adopting a One Health approach to mitigate the spread of resistance from food to humans.

## AUTHORS’ CONTRIBUTIONS

AMT, ZKK, and MHGK: Study concept and design. MHGK: Laboratory work and drafted the manuscript. ZKK and AMT: Data collection and statistical analysis. AMT and MHGK: Edited the manuscript. All authors have read and approved the final manuscript.
